# *Bandoniozyma* gen. nov., a Genus of Fermentative and Non-Fermentative Tremellaceous Yeast Species

**DOI:** 10.1371/journal.pone.0046060

**Published:** 2012-10-09

**Authors:** Patricia Valente, Teun Boekhout, Melissa Fontes Landell, Juliana Crestani, Fernando Carlos Pagnocca, Lara Durães Sette, Michel Rodrigo Zambrano Passarini, Carlos Augusto Rosa, Luciana R. Brandão, Raphael S. Pimenta, José Roberto Ribeiro, Karina Marques Garcia, Ching-Fu Lee, Sung-Oui Suh, Gábor Péter, Dénes Dlauchy, Jack W. Fell, Gloria Scorzetti, Bart Theelen, Marilene H. Vainstein

**Affiliations:** 1 Departamento de Microbiologia, Imunologia e Parasitologia, Universidade Federal do Rio Grande do Sul, Porto Alegre - RS, Brazil; 2 Centro de Biotecnologia, Universidade Federal do Rio Grande do Sul, Porto Alegre – RS, Brazil; 3 Centraalbureau voor Schimmelcultures Fungal Biodiversity Centre, Utrecht, The Netherlands; 4 Departamento de Bioquímica e Microbiologia, São Paulo State University, Rio Claro - SP, Brazil; 5 Coleção Brasileira de Micro-organismos de Ambiente e Indústria, Divisão de Recursos Microbianos-Centro Pluridisciplinar de Pesquisas Quimicas, Biologicas e Agricolas, Universidade Estadual de Campinas, Campinas – SP, Brazil; 6 Departamento de Microbiologia, Universidade Federal de Minas Gerais, Belo Horizonte – MG, Brazil; 7 Laboratório de Microbiologia Ambiental e Biotecnologia, Campus Universitário de Palmas, Universidade Federal do Tocantins, Palmas - TO, Brazil; 8 Instituto de Microbiologia Prof. Paulo de Goes, Universidade Federal do Rio de Janeiro, Rio de Janeiro - RJ, Brazil; 9 Department of Applied Science, National Hsinchu University of Education, Hsinchu, Taiwan; 10 Mycology and Botany Program, American Type Culture Collection, Manassas, Virginia, United States of America; 11 National Collection of Agricultural and Industrial Microorganisms, Faculty of Food Sciences, Corvinus University of Budapest, Budapest, Hungary; 12 Rosenstiel School of Marine and Atmospheric Science, University of Miami, Key Biscayne, Florida, United States of America; New York State Health Department and University at Albany, United States of America

## Abstract

**Background:**

Independent surveys across the globe led to the proposal of a new basidiomycetous yeast genus within the *Bulleromyces* clade of the Tremellales, *Bandoniozyma* gen. nov., with seven new species.

**Methodology/Principal Findings:**

The species were characterized by multiple methods, including the analysis of D1/D2 and ITS nucleotide sequences, and morphological and physiological/biochemical traits. Most species can ferment glucose, which is an unusual trait among basidiomycetous yeasts.

**Conclusions/Significance:**

In this study we propose the new yeast genus *Bandoniozyma*, with seven species *Bandoniozyma noutii* sp. nov. (type species of genus; CBS 8364^T^  =  DBVPG 4489^T^), *Bandoniozyma aquatica* sp. nov. (UFMG-DH4.20^T^  =  CBS 12527^T^  =  ATCC MYA-4876^T^), *Bandoniozyma complexa* sp. nov. (CBS 11570^T^  =  ATCC MYA-4603^T^  =  MA28a^T^), *Bandoniozyma fermentans* sp. nov. (CBS 12399^T^  =  NU7M71^T^  =  BCRC 23267^T^), *Bandoniozyma glucofermentans* sp. nov. (CBS 10381^T^  =  NRRL Y-48076^T^  =  ATCC MYA-4760^T^  =  BG 02-7-15-015A-1-1^T^), *Bandoniozyma tunnelae* sp. nov. (CBS 8024^T^  =  DBVPG 7000^T^), and *Bandoniozyma visegradensis* sp. nov. (CBS 12505^T^  =  NRRL Y-48783^T^  =  NCAIM Y.01952^T^).

## Introduction

Phylogenetic approaches to yeast systematics have revealed the polyphyletic nature of many yeast genera, emphasizing the need for a natural classification. As a consequence, several new genera have been described in an increasing effort to classify yeasts according to monophyletic clades with high bootstrap support [1], [2]. Following this approach, new genera were described in the Tremellales (Agaricomycotina, Basidiomycota), a large group of basidiomycetes that include basidiocarp-forming species, anamorphic yeast taxa and dimorphic fungi with complex life cycles bearing unicellular yeast phases [3], [4]. Some of the yeast genera in Tremellales are teleomorphic (i.e. sexual), such as *Auriculibuller*, *Bulleromyces*, *Cuniculitrema*, *Papiliotrema*, *Bulleribasidium* and *Kwoniella* [5]–[9], but anamorphic (i.e. asexual) genera have also been described. The *Luteolus* clade of the Tremellales gave rise to the anamorphic genera *Derxomyces* and *Hannaella* for distinct clades represented by *Bullera mrakii* and *B. sinensis*, respectively [10], while Takashima et al. [11] emended *Dioszegia* and transferred *Cryptococcus hungaricus* to this genus, and Wang et al. [12] described *Mingxiaea* to accommodate the anamorphic species in the *Bulleribasidium* clade. According to Statzell-Tallman et al. [9], the Tremellales are a large and weakly structured group of basidiomycetes, composed of distinct clades. One of these clades, *Bulleromyces*, is poorly supported in both D1/D2 and ITS trees, and thus deserves a detailed study [13], [14].

This manuscript deals with the description of a new genus of basidiomycetous yeast species in the *Bulleromyces* clade of the Tremellales. Various surveys by a number of independent researchers across the globe resulted in a collection of strains obtained from different countries and substrates. Most strains are capable of glucose fermentation, which is an uncommon biochemical trait among the basidiomycetous yeasts. Using molecular phylogenetic approaches we propose the new monophyletic genus *Bandoniozyma* gen. nov., and seven new species: *Bandoniozyma noutii* sp. nov. (type species of the genus), *Bandoniozyma aquatica* sp. nov., *Bandoniozyma complexa* sp. nov., *Bandoniozyma fermentans* sp. nov., *Bandoniozyma glucofermentans* sp. nov., *Bandoniozyma tunnelae* sp. nov., and *Bandoniozyma visegradensis* sp. nov.

## Materials and Methods

### Yeast Isolation and Maintenance

The localities, substrates of isolation, MycoBank numbers and GenBank accession numbers are summarized in Table 1 (see Information S1 for details).

**Table 1:** List of strains and DNA sequences used in this study.

Species	MycoBanknumber (MB)	Strain *	Origin	GeneBank accession number	
				D1/D2	ITS
*Bandoniozyma noutii*	MB 563852				
		CBS 8364^T^ (DBVPG 4489^ T^)	Exudate of *Eriobotrya japonica*(Rosaceae), Tijuca Forest, RJ, Brazil	AF444700	AF444391
		CBS 8365 (DBVPG 4490)	Exudate of *Eriobotrya japonica*(Rosaceae), Tijuca Forest, RJ, Brazil	AF444701	AF444392
		CBS 8368 (DBVPG 4499)	Flower of *Pimenta dioica* (Myrtaceae),Pau da Fome, Pedra Branca, RJ, Brazil	AF444704	AF444395
*B. aquatica*	MB 563857				
		UFMG-DH4.20^T^ (CBS 12527^T^,ATCC MYA-4876^ T^)	Freshwater in a Lake (Dom Helvécio)from Parque Estadual do Rio Doce,MG, Brazil	JN979992	JN790616
*B. complexa*	MB 801195				
Group I		CBS 11570^T^ (ATCC MYA-4603^T^, MA28a^T^)	Air from timber factory Cachoeirado Sul, RS, Brazil	GU321090	GU321089
		MA68d	Air from timber factory, Cachoeirado Sul, RS, Brazil	GU321092	GU321091
Group II		CBS 12531 (CBMAI 1003 )	Aluminum screw with signs of corrosionfrom an energy transmission tower,Suzano, SP, Brazil	FJ986613	JQ070069
		BD 143	Aluminum screw with signs ofcorrosion from an energy transmissiontower, Suzano, SP, Brazil	–	–
		BD 149	Aluminum screw with signs ofcorrosion from an energy transmissiontower, Suzano, SP, Brazil	–	–
		IMUFRJ 51948	Leaf of *Neoregelia cruenta*(Bromeliaceae), Restinga deMaricá, RJ, Brazil	FN424103	FN424103
Group III		CBS 12398 (BCRC 23285,PL04)	Pineapple, Hsinchu, Taiwan	FJ527161	HQ623538
		UFMG-LR3.11	Lago Rico Lake, Parque Estadualdo Cantão,TO, Brazil	–	–
		UFMG-LD2.09	Lago de Dentro Lake, ParqueEstadual do Cantão,TO, Brazil	–	–
		UFMG-LD3.02	Lago de Dentro Lake, ParqueEstadual do Cantão,TO, Brazil	JN997534	JN997533
*B. fermentans*	MB 563855				
		CBS 12399^T^ (BCRC 23267^T^,NU7M71^T^)	Unidentified mushroom, Beinan,Taitung, Taiwan	HM461720	HQ623541
*B. glucofermentans*	MB 563856				
		CBS 10381^T^ (NRRL Y-48076^T^,ATCC MYA-4760^T^, BG 02-7-15-015A-1-1^T^)	Gut of *Amphix laevigatus*(Coleoptera: Endomychidae),Panama	AY520334	JN381033
		NRRL Y-48077 (ATCC MYA-4761, BG 02-7-16-015A-1-1)	Gut of *Canthon* sp. (Coleoptera:Scarabaeidae), Panama	AY520385	JN381034
*B. tunnelae*	MB 563853				
		CBS 8024^T^ (DBVPG 7000^ T^)	Nail, Finland	AF444715	AF444453
		CBS 6123 (DBVPG 6993)	Unknown substrate, Finland	AF444687	AF444333
		CBS 6024 (DPCPG 6992;PYCC 4857)	Unknown substrate, Finland	AF444714	AF444452
*B. visegradensis*	MB 563854				
		CBS 12505^T^, (NRRL Y-48783^T^,NCAIM Y.01952^T^)	Exudate of *Quercus cerris*, Hungary	GU195658	HQ660084

* Type strain, T.

ATCC  =  American Type Culture Collection, USA.

BCRC  =  Bioresources Collection and Research Center,Taiwan.

CBMAI  =  Brazilian Collection of Environmental and Industrial Microorganisms, Brazil.

CBS  =  Centraalbureau voor Schimmelcultures Fungal Biodiversity Center, The Netherlands.

DBVPG  =  Dipartimento di Biologia Vegetale dell'Universita di Perugia Industrial Yeasts Collection, Italy.

IMUFRJ  =  Instituto de Microbiologia Prof. Paulo de Góes - Federal University of Rio de Janeiro Culture.

NCAIM  =  National Collection of Agricultural and Industrial Microorganisms, Hungary.

Collection, Brazil.

NRRL  =  ARS (Agricultural Research Service) Culture Collection, USA.

UFMG  =  Universidade Federal de Minas Gerais Culture Collection, Brazil.

### Phenotypic Characterization

Morphological and biochemical/physiological characterization of the isolates was performed according to Kurtzman et al. [15] and Barnett et al. [16].

### DNA Sequencing and MSP-PCR (Microsatellite-primed PCR) Fingerprinting

The D1/D2 domain of the large subunit (26S) rRNA gene was sequenced as described by Kurtzman and Robnett [17]. The ITS region (ITS1, 5.8S rRNA gene and ITS2) was amplified and sequenced as described by Péter et al. [18]. Alignments and phylogenetic trees were constructed with MEGA 5 [19], using the neighbor joining method with bootstrap analysis based on 10,000 random samplings. MSP-PCR fingerprinting followed the protocols described in Sampaio et al. [20], and used the primers (GTG)_5_ and M13. MSP-PCR profiles with each primer were repeated twice for inference of reproducibility. Gel electrophoresis images were acquired with the GelDoc XR System software (Bio-Rad).

### Nomenclature

The electronic version of this article in Portable Document Format (PDF) in a work with an ISSN or ISBN will represent a published work according to the International Code of Nomenclature for algae, fungi, and plants, and hence the new names contained in the electronic publication of a PLOS ONE article are effectively published under that Code from the electronic edition alone, so there is no longer any need to provide printed copies.

In addition, new names contained in this work have been submitted to MycoBank from where they will be made available to the Global Names Index. The unique MycoBank number can be resolved and the associated information viewed through any standard web browser by appending the MycoBank number contained in this publication to the prefix http://www.mycobank.org/MB. The online version of this work is archived and available from the following digital repositories: PubMed Central; LOCKSS.

## Results and Discussion

### Proposal of New Genus

**Figure 1:**
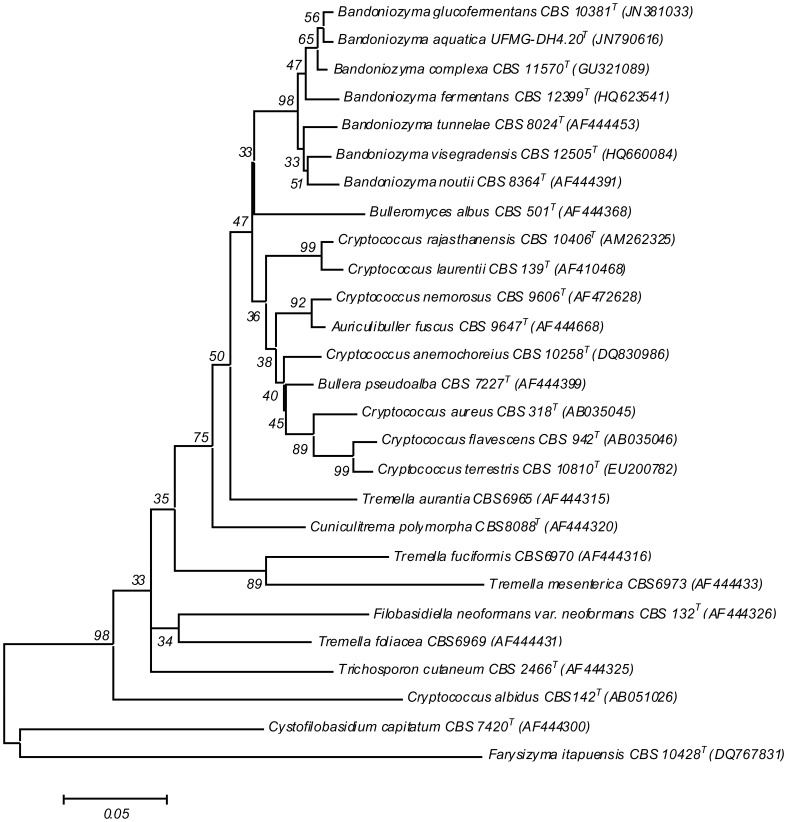
ITS tree showing the phylogenetic relationship among the Tremellomycetes, with emphasis on the *Bandoniozyma* clade, obtained by neighbor-joining analysis using Mega 5.0. Most species belong to the order Tremellales, with the exception of *Cystofilobasidium capitatum* (Cystofilobasidiales), *Cryptococcus albidus* (Filobasidiales), *Trichosporum cutaneum* (Trichosporonales), and the outgroup *Farysizyma itapuensis* (Ustilaginales). The numbers given on the branches are the frequencies with which a given branch appeared in 10,000 bootstrap replications. Bar, substitutions per nucleotide position.

Data analysis of partial rDNA sequences allowed the recognition of a new clade of basidiomycetous yeast species that originated from different geographic locations (Table 1, Fig. 1). Most of the species within this clade are capable of glucose fermentation, and some species can ferment other sugars as well (Table 2, Table S1). Phylogenetically, the clade is located in the *Bulleromyces* clade of Tremellales sensu Scorzetti et al. [14], and is surrounded by species belonging to *Cryptococcus*, a well-recognized polyphyletic genus of the class Tremellomycetes [21]. Although fermentative ability is an uncommon feature in basidiomycetous yeasts, it has been reported in species of *Mrakia*, *Mrakiella* and *Xanthophyllomyces* [22]–[24], and in *Filobasidium capsuligenum* [25]. The current diagnosis of the genus *Cryptococcus* accommodates the inclusion of fermentative isolates, but there are no currently recognized *Cryptococcus* species with this ability [21]. *Cryptococcus aquaticus* was once considered a fermentative *Cryptococcus*, but it was transferred to the genus *Mrakiella*, as *M. aquatica* [26]. As classification of yeasts in monophyletic taxa is highly desirable, the proposal of a new genus for this clade is justified rather than describing new yeast species of *Cryptococcus*, which would increase the polyphyletic nature of this genus. As the generic name *Cryptococcus* will be confined to the *C. neoformans* clade [27], we propose a new monophyletic genus, *Bandoniozyma*, to accommodate those species. Hitherto, the new genus contains only anamorphic yeasts, but it cannot be ruled out that sexual stages could be discovered in the future.

**Table 2:** Selected physiological/biochemical test responses for differentiation of the newly proposed *Bandoniozyma* species. The full data can be seen in Table S1.

Test responses*	*B. noutii*	*B. aquatica*	*B. complexa*	*B. fermentans*	*B. glucofermentans*	*B. tunnelae*	*B. visegradensis*
***Fermentation***							
D-Glucose	+	+	V	+	D	+,D	−
***Carbon sources***							
D-Galactose	+	+	+	+	+	+	−
L-Arabinose	+	+	+	+	+	+	−
D-Arabinose	+	−	+,D	W	+	+	+,D
Melibiose	+	−	+	−	+	−	−
Lactose	−	+	V	−	−,W	V	−
Raffinose	+	−	+	−	+	V	−
Starch	−	−	V	W	−	V	+,W
Glycerol	+	+	V	−	+	+,D	+,D
Erythritol	−	+	V	−	+	+,D	−
Xylitol	+	+	V	W	+	+,D	D,V
L-Arabinitol	+	+	V	+	+	+,D	−
Galactitol	V	+	V	+	W	−	−
DL-Lactate	+	−	+,D	W	−	+	+
Citrate	+	V	V	W	W	+	+
***Nitrogen sources***							
Nitrite	+,W	−	+	W	−	−	W
Ethylamine	+	+	+	+	+	V	−
***Vitamin requirements***							
Vitamin free	nd	+	+	+	−	−	−
***Other tests***							
0.01% cycloheximide	+	+	V	+	+	V	+
50%D-Glucose	+	−	V	−	−	V	−
Starch formation	+	W	+	−	+	+,W	+

* Test results: +, positive; D, delayed positive; W, weak; −, negative; V, variable; nd, not determined.

The proposed new genus forms a sister clade to the *Auriculibuller*/*C. laurentii*/*C. flavescens* complex. According to Scorzetti et al. [14], the *Bulleromyces* clade is weakly supported in both D1/D2 and ITS trees, and requires a detailed study of its biology and molecular systematics. Several new yeast species have been described recently in the *Bulleromyces* clade [28]–[32]. Additionally, genetic diversity among the strains previously identified as *C. laurentii* has been recognized [33], [34]. Finally, the large number of new species in the presently discussed fermentative basidiomycetous yeast group emphasizes the need for a taxonomic revision of the Tremellales.

### Proposal of New Species

The species in the *Bandoniozyma* clade are mainly separated from each other based on the ITS sequences (Table 1, Figure 1), while the D1/D2 sequences of the 26S rDNA were less informative (showed 2 to 13 nucleotide substitutions). Pairwise comparisons between the species show that most of them have 14 to 29 ITS nucleotide substitutions with each other, except in the case of the comparisons between *Bandoniozyma glucofermentans* and *Bandoniozyma aquatica*, which will be discussed later. Intraspecific ITS variability is less than 2 nucleotide substitutions (viz., *Bandoniozyma noutii* CBS 8364^T^, CBS 8365 and CBS 8368; *Bandoniozyma tunnelae* CBS 8024^T^, CBS 6024 and CBS 6123; and *B. glucofermentans* CBS 10381^T^ and ATCC MYA-4761), unless in the case of *B. complexa* (CBS 11570^T^, MA68d, CBS 12531, IMUFRJ 51948, CBS 12398 and UFMG-LD3.02), whose strains have 2 to 7 nucleotide substitutions in the pairwise comparisons. The biochemical/physiological profiles of all the species are given in Table 2 and Table S1. All the species assimilate D-glucose, D-xylose, L-rhamnose, sucrose, maltose, a-a-trehalose, cellobiose, D-glucitol, D-manitol, myo-inositol, D-gluconate, succinate, L-lysine, grow at 25°C and are DBB positive. None of the species ferment galactose, and assimilate methanol or nitrate.

The genus is comprised of two clearly separated groups of species (Figure 1). The first group is composed of *Bandoniozyma tunnelae*, *Bandoniozyma noutii* and *Bandoniozyma visegradensis*. The latter two species are represented by strains obtained mainly from plant exudates. It is significant to note that *B. noutii* is able to ferment sucrose and raffinose, which are two of the most common sugar components of plant exudates [35], [36]. While most species in the *Bandoniozyma* genus originated from tropical/subtropical areas and environmental samples, *B. tunnelae* and *B. visegradensis* are from Finland and Hungary, respectively, and *B. tunnelae* was obtained from a human-related substrate (Table 1).

*Bandoniozyma glucofermentans* was isolated from the gut of insects, while *Bandoniozyma fermentans* was isolated from an unidentified mushroom (Table 1). The type strain of *B. glucofermentans* was isolated from *Amphix laevigatus* (Coleoptera: Endomychidae), a mycophagous beetle [37]. Although an additional strain of *B. glucofermentans* was found from *Canthon* sp. (Coleoptera: Scarabaeidae), which is usually copro-necrophagous, some *Canthon* species have a generalist feeding behavior [38]. The hypothesis that *B. glucofermentans* and *B. fermentans* take part in the beetle/mushroom/yeast interaction model should be further investigated to evaluate if additional fermentative basidiomycetous yeasts occur in mushroom related sources. *B. glucofermentans* is closely related to *Bandoniozyma aquatica*, which differs from the former in the ITS sequence (5 substitutions), assimilation of D-arabinose, melibiose, raffinose, and growth in vitamin-free medium (Table 2). *B. aquatica*, like other *Bandoniozyma* strains discussed below, was isolated from lake water, suggesting that these yeasts can also be dispersed by water. The lake where this yeast was isolated is surrounded by Atlantic Rain Forest, and its occurrence in this aquatic environment could be considered the result of a run-off from plant or insects of this forest.

*Bandoniozyma complexa* is represented by 10 strains isolated from different substrates and geographical regions (Table 1). These strains group in three different MSP-PCR fingerprinting profiles with primers M13 (Figure S1) and GTG_5_ (data not shown). MSP-PCR fingerprinting is a methodology that displays whole genome profiles, and isolates that belong to the same species usually present identical or similar fingerprints [39]. *B. complexa* group I differs from group II by 7 nucleotide substitutions in the ITS sequences, assimilation of starch, glycerol, erythritol, L-arabinitol, galactitol, citrate and growth in the presence of 0.01% cycloheximide, while group III differs from group I by 4 ITS nucleotide substitutions, glucose fermentation and nitrite assimilation, and from group II by 3 substitutions, glucose fermentation, assimilation of starch, glycerol, erythritol, citrate, nitrite and growth in the presence of 0.01% cycloheximide (data not shown). In particular, glucose fermentation is negative for strains in group III and strongly positive in less than three days for groups I and II. The biochemical tests were confirmed in two different laboratories. Group III strain CBS 12398 does not form pseudohyphae nor true hyphae on Dalmau plate culture on corn meal agar, while strains belonging to groups I and II do. The different MSP-PCR profiles with two independent primers and phenotypic traits suggest these groups are separate species, but D1/D2 and ITS sequencing could not clearly differentiate them. It is possible that the strains in this complex have recently diverged, and the ITS sequences may not be the best molecular marker for differentiation of these putative species. Attempts were made to sequence other genes (EF1-alpha and cytochrome B), but sequencing problems prevented conclusive results (Information S2).

*Bandoniozyma complexa* group I strains were isolated from air from a timber factory in South Brazil, which dealt mostly with wood of *Pinus* spp., but also processed *Ficus* and *Eucalyptus* trees (Table 1), suggesting that it may have been air-dispersed from a plant-related origin. *B. complexa* group III strain CBS 12398 was isolated from pineapple in Taiwan, while three other strains from this group were isolated from lake water within an Amazon Forest ecosystem in Northern Brazil (Table 1, Figure S1), thus suggesting dispersion from a primary plant-related substrate. The fact that *B. complexa* group III isolates were found in two geographically distant tropical countries indicates that this group may have a wide distribution in tropical environments.

*Bandoniozyma complexa* group II strains CBS 12531, BD143 and BD149 were isolated from a biofilm associated with a corroded aluminum screw from an energy transmission tower in Southeast Brazil (Table 1), which contained several other species of filamentous fungi and yeasts [40]. Strain CBS 12531^T^ was shown to be conspecific with strain IMUFRJ 51948 by means of D1/D2 and ITS sequences, and both were isolated from geographically close locations in Brazil. Considering that strain IMUFRJ 51948 was obtained from a bromeliad, and that many *Bandoniozyma* strains were isolated from plant substrates and lake water, it could be suggested that association of *B. complexa* group II with the corrosion biofilm occurred after dispersion from a primary environmental source, although a role in biofilm formation cannot be excluded. Fungal influenced corrosion of a variety of materials, such as metals, minerals and concrete, is well known [41], [42], and there are reports of isolation of yeasts from substrates damaged by corrosion [43], [44].

**Figure 2:**
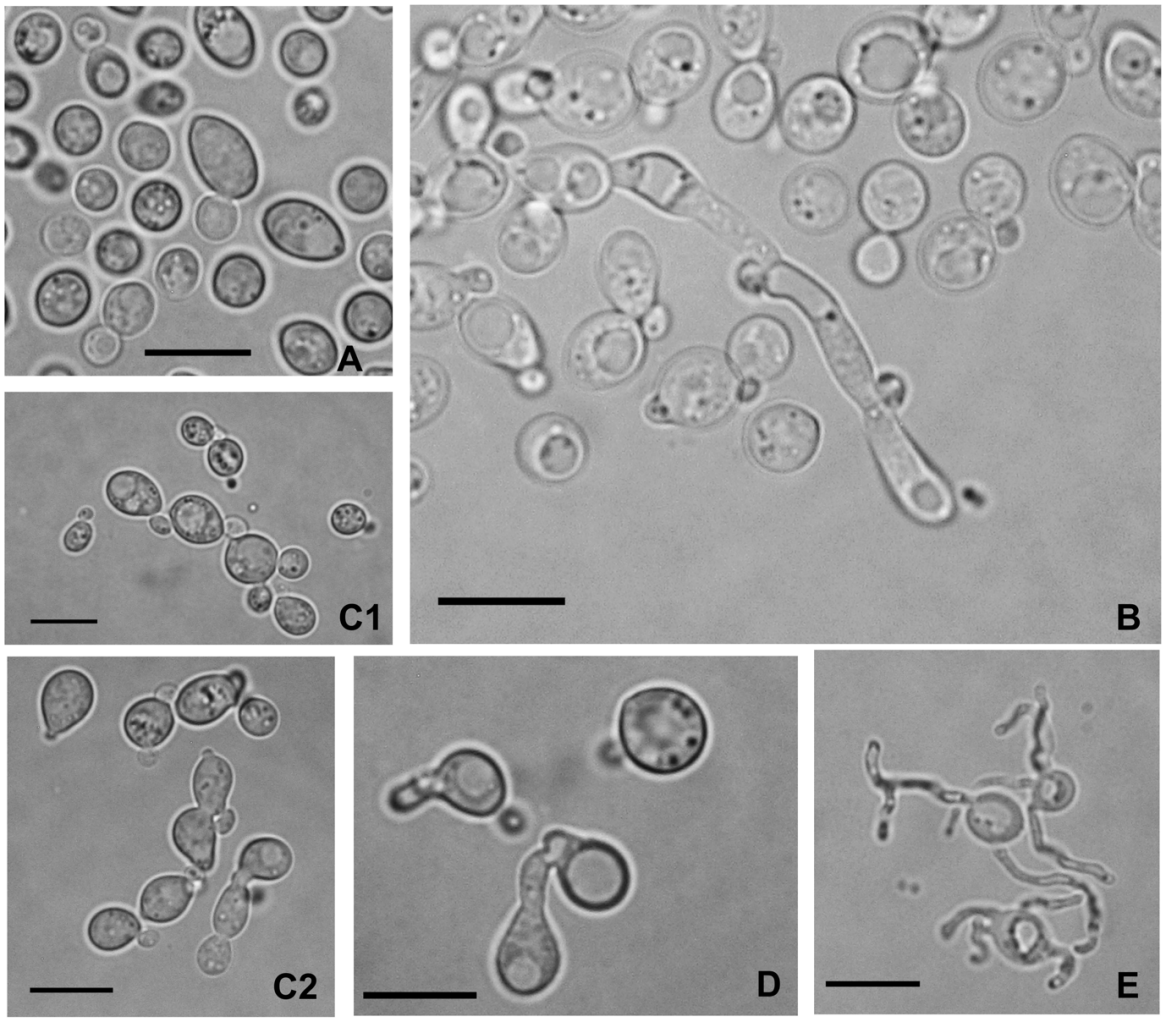
*Bandoniozyma visegradensis* CBS 12505^T^ a) Growth on 5% malt extract agar, 3 days, 25°C, b) Septate hypha in 5% malt extract broth, 19 days, 25°C, c) Curved buds connecting independent cells (c.1) or mother cell bud pairs (c.2), YM agar, 10 days, 25°C, d) Conjugating cells, YM agar, 10 days, 25°C, e) Conjugation tubes, 2% malt extract agar, 18 days, 6°C. Bar = 10 µm.

Although a sexual stage could not be observed for any of the proposed *Bandoniozyma* species, *B. visegradensis* strain CBS 12505^T^ formed septate hyphae and presented conjugating cells and tubes (Figure 2). Studies concerning the reproductive behaviour of *B. visegradensis* and isolation of more strains of this species will probably improve the current circumscription of this group of yeasts.

We hypothesize that the fermentation ability of the *Bandoniozyma* cluster was acquired after its separation from the non-fermentative *C. laurentii*/*C. flavescens* clade. Secondarily, *B. complexa* group III and *B. visegradensis* may have lost the fermentative capacity that is typical of the whole group. As glucose fermentation in yeasts is a two-step reaction mediated by the enzymes pyruvate decarboxylase and alcohol dehydrogenase [45], the alternative hypothesis of multiple independent origins for the fermentative ability of the species in this group is highly improbable. The fermentative capabilities of some *Bandoniozyma* species (i.e. *B. noutii*, *B. tunnelae*, *B*. *fermentans*, *B. glucofermentans*, *B. aquatica*, *B*. *complexa* groups I and II) are unusual for basidiomycetous yeasts due to the strong glucose fermentation at 25–28°C, which is comparable to some ascomycetous species that vigorously ferment sugars.

We isolated several fermentative basidiomycetous strains from plant substrates (leaf surface, exudates and flowers) and mushroom, in addition to other possible secondary substrates, associated with the dispersion of these yeasts, such as insects, air, water, corroded aluminum screw, and human nail. These substrates were mostly sampled in tropical/subtropical areas (Brazil, Panama and Taiwan), but a temperate country (Finland) also yielded fermentative strains (*B. tunnelae* strains CBS 8024^T^, CBS 6024 and CBS 6123). As the sampling areas are globally distributed, it seems that the main premise for the presence of these yeasts is the availability of simple sugars as substrates for fermentation. We foresee that more fermentative basidiomycetous yeast strains will be reported as new simple sugar-containing substrates are sampled.

### *Bandoniozyma* Boekhout, Valente, Pagnocca, Rosa, Lee, Suh, Blackwell, Péter, & Fell gen. nov

Valente et al. 2012, gen.nov. [urn:lsid:imycobank.org:names: MB 563851.

Budding cells are globose, subglobose, ovoid or ellipsoidal. Asexual reproduction is by polar or multilateral budding. Colonies are white, cream- colored to yellowish, smooth and butyrous or mucoid. Hyphae or pseudohyphae may be present. Clamp connections may be present. Sexual reproduction was not observed. Ballistoconidia are not produced. Fermentation of glucose is generally present. Diazonium blue B and urease reactions are positive. Growth on *myo*-inositol and D-glucuronate are positive, but growth on nitrate is negative. Starch-like compounds are generally formed. Type species: *Bandoniozyma noutii.*

Etymology: The genus is named in honour of Robert (Bob) J. Bandoni, University of British Columbia, who dedicated his life to the study of the Tremellales.

The following species are accepted in the genus and can be differentiated by ITS sequencing and the biochemical/physiological tests included in Table 2.

### 1. *Bandoniozyma noutii* Boekhout, Fell, Scorzetti & Theelen sp. nov

Valente et al. 2012, sp. nov. [urn:lsid:imycobank.org:names: MB 563852.

Etymology: The specific epithet *noutii* refers to Robert (Rob) Nout, investigator of traditionally fermented foods from South America, Africa and Asia.

**Figure 3:**
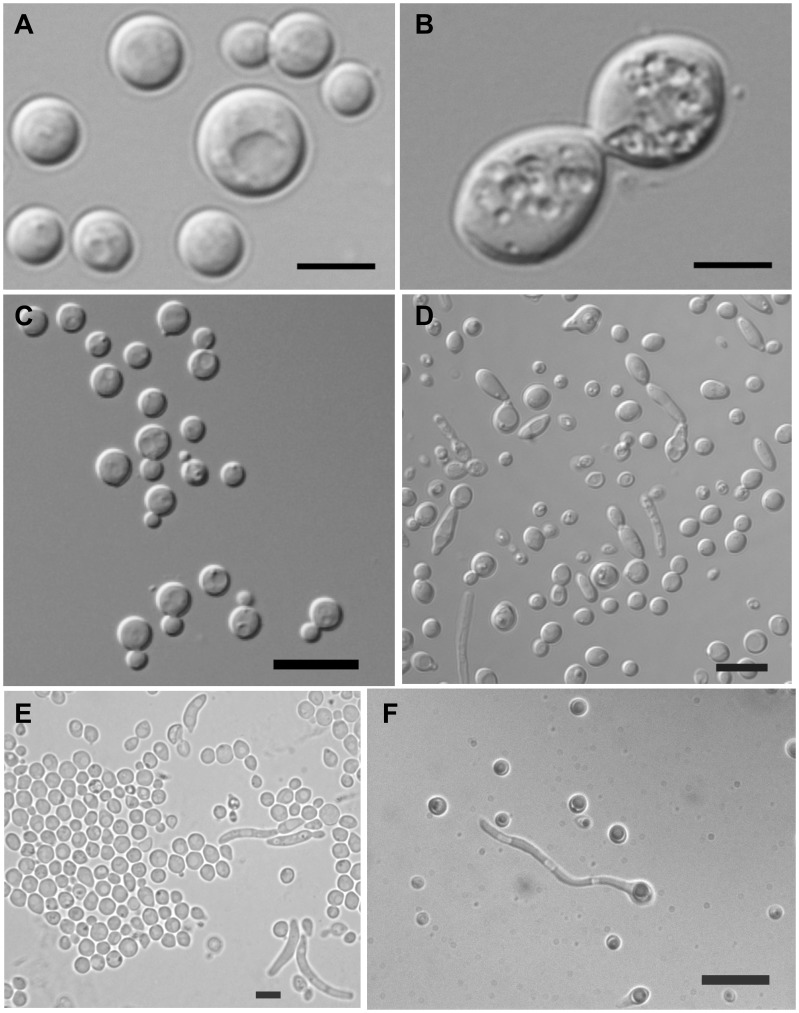
Cell morphologies: a) *Bandoniozyma noutii* CBS 8364^T^ after 7 days in YM broth at 25°C. Bar = 10 µm, b) *Bandoniozyma tunnelae* CBS 8024^T^ after 7 days in YM broth at 25°C. Bar = 10 µm, c) *Bandoniozyma fermentans* CBS 12399^T^ grown in YMA for 3 day at 25 °**C. Bar = 10 µm, d)** ***Bandoniozyma glucofermentans*** **CBS 10381^T^ after 7 days in YM broth at 25°C. Bar = 10 µm, e)** ***Bandoniozyma aquatica*** **CBS 12527^T^ grown in YEPD broth for 3 days at 25°C. Bar = 10 µm, and f)** ***Bandoniozyma complexa*** **CBS 11570^T^ grown in YEPD broth for 3 days at 25°C. Bar = 10 µm.**

After growth for 7 days in 2% glucose medium at 25°C, a sediment and film are formed. Cells are ovoid, subglobose to globose, 4–6×4–5 µm, usually with polar budding but also with multilateral budding, with buds that may adhere into short chains (Figure 3a). Pseudohyphae are present and fall apart in filaments that measure 28–50(−70)×2–4 µm, and eventually become somewhat thick-walled, irregularly broadened or somewhat broadened on one side. After 5 days on 5% malt extract agar at 25°C, colonies are 10 mm in diameter, convex, grayish cream-beige, shiny, strongly mucoid, smooth, and with an entire margin that may form sectors. Cells measure 3.3–7.8×3–7 µm. Under a cover glass filaments and hyphae occur with cells measuring 8–40×2–2.5 µm. On Dalmau plate on yeast morphology agar, pseudohyphae occur with cells measuring 8–40×2–2.5 µm. Sexual reproduction is absent. Mixing the three available strains on potato dextrose agar, oat meal agar, corn meal agar, malt extract agar, glucose-yeast extract agar, and yeast extract malt extract agar did not show any indication of a mating reaction. Physiological/biochemical test responses can be seen in Table 2 and Table S1.

Strains investigated: CBS 8364^T^ ( = DBVPG 4489^T^), CBS 8365 ( = DBVPG 4490), from exudate of *Eriobotrya japonica* (Rosaceae), Tijuca Forest, RJ, Brazil; and CBS 8368 ( = DBVPG 4499), from Flower of *Pimenta dioica* (Myrtaceae), Pau da Fome, Pedra Branca, RJ, Brazil, all isolated by G. Capriotti, DBVPG. The type strain has been deposited in Centraalbureau voor Schimmelcultures (CBS) and DBVPG Industrial Yeasts culture collections.

### 2. *Bandoniozyma aquatica* Brandão, Valente, Pimenta & Rosa sp. nov

Valente et al. 2012, sp. nov. [urn:lsid:imycobank.org:names: MB 563857.

Etymology: The specific epithet *aquatica* refers to the habitat (freshwater) from which this species was isolated.

After growth in yeast extract-malt extract-peptone-glucose broth at 25°C for 3 days, the cells are globose to subglobose, 3.1–4.7×2.8–4.9 µm, and occur singly or in pairs (Figure 3e). Asexual reproduction is by multilateral budding. Sediment is present. After 7 days at 25°C on yeast extract-malt extract-peptone-glucose agar, streak cultures are cream, butyrous, rugose, and glistening. On Dalmau plate cultures on corn meal agar after 10 days at 25°C, pseudohyphae are formed. Sexual reproduction was not observed. Ballistoconidia are not produced. Physiological/biochemical test responses can be seen in Table 2 and Table S1.

Strain investigated: UFMG-DH-4.20^T^ ( = CBS 12527^T^, ATCC MYA-4876^T^), from a freshwater sample, Ecological Reserve of Rio Doce, Brazil, isolated by L. Brandão. The type strain has been deposited in Universidade Federal de Minas Gerais (UFMG), American Type Culture Collection (ATCC) and Centraalbureau voor Schimmelcultures (CBS) culture collections.

### 3. *Bandoniozyma complexa* Landell, Pagnocca, Sette, Passarini, Garcia, Ribeiro, Lee, Brandão, Rosa & Valente sp. nov

Valente et al. 2012, sp. nov. [urn:lsid:imycobank.org:names: MB 801195.

Etymology: The specific epithet *complexa* refers to the phenotypic and genotypic variation observed in the strains presently maintained in the species.

In glucose-peptone-yeast extract broth or yeast extract-malt extract-peptone-glucose after 3 to 4 days at 25°C, the asexual cells are globose to subglobose and ovoid, and occur singly or in pairs, 1.8–4.6×2.0–4.3 µm, and occur singly or in pairs (Figure 3f). After 1 week on glucose-peptone-yeast extract agar or yeast extract-malt extract-peptone-glucose agar at 25°C, the streak culture is white, creamy, smooth, butyrous and glistening. After 3 weeks in Dalmau plate culture on cornmeal agar, pseudohyphae and true hyphae formation are variable. Sediment is formed. Asexual reproduction is by multilateral budding. Sexual reproduction was not observed. Mixing the available strains on corn meal agar did not show any indication of a mating reaction after 6 days at 25°C. On yeast extract-malt extract-peptone-glucose agar after 3 days at 25°C, colonies are smooth, mucoid to butyrous, glistening and cream-colored, and have an entire margin. Ballistoconidia are not produced. Physiological/biochemical test responses can be seen in Table 2 and Table S1.

Strains investigated: CBS 11570^T^ ( = ATCC MYA-4603^T^, MA28a^ T^) and MA68d, from air samples in Rio Grande do Sul, Brazil, isolated by J. Crestani; CBS 12531 ( = CBMAI 1003), from a corroded screw from an energy transmission tower in Suzano, SP, Brazil, isolated by M. Passarini; IMUFRJ 51948, from the bromeliad *Neoregelia cruenta* in Rio de Janeiro, Brazil, isolated by K. Garcia; CBS 12398 ( = BCRC 23285, PL04), from pineapple, Hsinchu, Taiwan, isolated by C-F Lee; UFMG-LR3.11, from freshwater of Lago Rico Lake, Parque Estadual do Cantão,TO, Brazil; UFMG-LD2.09 and UFMG-LD3.02, from freshwater of Lago de Dentro Lake, Parque Estadual do Cantão,TO, Brazil, all isolated by R. Pimenta & L.R. Brandão. The type strain has been deposited in American Type Culture Collection (ATCC) and Centraalbureau voor Schimmecultures (CBS) culture collections.

### 4. Bandoniozyma fermentans Lee sp. nov

Valente et al. 2012, sp. nov. [urn:lsid:imycobank.org:names: MB 563855.

Etymology: The specific epithet *fermentans* refers to the ability of the species to ferment glucose and other sugars.

After growth in yeast extract-malt extract-peptone-glucose broth at 25°C for 3 days, the cells are globose to subglobose, 1.9–4.4×2.3–4.6 µm, and occur singly or in pairs (Figure 3c). Asexual reproduction is by multilateral budding. Sediment is present. After 7 days at 25°C on yeast extract-malt extract-peptone-glucose agar, streak cultures are creamy, butyrous, smooth, and glistening. On Dalmau plate cultures on corn meal agar after 10 days at 25°C, neither pseudohyphae nor true hyphae are formed under the cover glass. Sexual reproduction was not observed. Ballistoconidia are not produced. Physiological/biochemical test responses can be seen in Table 2 and Table S1.

Strain investigated: CBS 12399^T^ ( = BCRC 23267^T^, NU7M71^T^), from fruiting body of an unidentified mushroom, Beinan, Taitung, Taiwan, isolated by C-F Lee. The type strain has been deposited in Bioresources Collection and Research Center (BCRC), Food Industry Research and Development Institute, Taiwan; and Centraalbureau voor Schimmelcultures (CBS) culture collections.

### 5. *Bandoniozyma glucofermentans* Suh & Blackwell sp. nov

Valente et al. 2012, sp. nov. [urn:lsid:imycobank.org:names: MB 563856.

Etymology: The specific epithet *glucofermentans* refers to the character of the species to ferment glucose.

In yeast extract-malt extract-peptone-glucose broth after 7 days at 25°C, cells are globose, subglobose, or ovoid, 2.5–5.0×2.5–6.5 µm, and occur singly or in pairs (Figure 3d). Pseudohyphae are present. On yeast extract-malt extract-peptone-glucose agar after 7 days at 25°C, colonies are cream colored, smooth, mucoid with a slightly filamentous edge. After 10 days of growth on Dalmau plate culture on cornmeal agar at 25°C, pseudohyphae and true hyphae are present. Aerobic growth is white to cream colored with a slightly fuzzy margin. Sexual reproduction was not observed. Sediment is formed. Ballistoconidia are not produced. Physiological/biochemical test responses can be seen in Table 2 and Table S1.

Strains investigated: CBS 10381^T^ ( = ATCC MYA-4760^T^, NRRL Y-48076^T^, BG 02-7-15-015A-1-1^T^), from the gut of *Amphix laevigatus* (Coleoptera: Endomychidae), Barro Colorado Island, Panama; and ATCC MYA-4761 ( = NRRL Y-48077, BG 02-7-16-015A-1-1), from the gut of *Canthon* sp. (Coleoptera: Scarabaeidae), Barro Colorado Island, Panama, all isolated by S-O Suh and M. Blackwell. The type strain has been deposited in American Type Culture Collection (ATCC), Centraalbureau voor Schimmelcultures (CBS), and ARS culture collections (NRRL).

### 6. *Bandoniozyma tunnelae* Boekhout, Fell, Scorzetti & Theelen sp. nov

Valente et al. 2012, sp. nov. [urn:lsid:imycobank.org:names: MB 563853.

Etymology: The specific epithet *tunnelae* refers to Dr. E. Tunnela, Finland, who isolated the strains.

After growth for 7 days in 2% glucose medium at 25°C, a sediment is formed, cells are ellipsoidal, ovoid, subglobose to globose, 5–9×2.5–7 µm (Figure 3b), usually with polar budding but also with multilateral budding, with clavate budding cells that may adhere into short chains; with pseudohyphae that fall apart in filaments that measure 28–50(−70)×2–4 µm, eventually becoming somewhat thick-walled, irregularly broadened or somewhat broadened on one side. After 5 days on 5% malt extract agar at 25°C colonies are 25–35 mm in diameter, flat to somewhat raised, cream-colored, shiny, mucoid, smooth, and with an entire to somewhat eroded margin. Cells are ovoid, subglobose to globose, 3.8–5.5×3.3–6 µm, with polar to multipolar budding. Under a cover glass filaments and hyphae occur with cells that measure 20–70×2–5 µm. On Dalmau plate on yeast morphology agar extensive hyphae and loosely branched pseudohyphae occur that laterally form blastoconidia near the septa, with cells measuring 15–30×2–3×4–7 µm; usually broadened at one end and remain catenulate; the broadened part may give rise to globose to ellipsoidal thick-walled chlamydospore-like cells that may release through endosporulation. Sexual reproduction is absent. Mixing the three available strains on potato dextrose agar, oat meal agar, corn meal agar, malt extract agar, glucose-yeast extract agar, and yeast extract malt extract agar did not show any indication of a mating reaction. The whole cell hydrolyzates of CBS 6024 contain glucose (main), galactose, mannose, xylose, arabitol, mannitol and glucuronic acid. Physiological/biochemical test responses can be seen in Table 2 and Table S1.

Strains investigated: CBS 8024^T^ ( = DBVPG 7000^T^), from human nail in Finland; CBS 6024 ( = DBVPG 6992, PYCC 4857), from unknown source; and CBS 6123 ( = DBVPG 6993), from unknown source, all isolated by E. Tunnela. The type strain has been deposited in Centraalbureau voor Schimmelcultures (CBS) and DBVPG Industrial Yeasts culture collections.

### 7. *Bandoniozyma visegradensis* Péter & Dlauchy sp. nov

Valente et al. 2012, sp. nov. [urn:lsid:imycobank.org:names: MB 563854.

Etymology: The specific epithet *visegradensis* refers to Visegrád, Hungary, the place where the type strain was isolated.

After 3 days on 5% malt extract agar at 25°C, the streak culture is mucoid, cream-colored to yellowish, smooth, slightly raised and glistening. The margin is entire. Cells are formed by multilateral budding, subspheroid, ovoid or ellipsoid, 3–9×4–13 µm, and occur singly and in pairs (Figure 2a). In 5% malt extract after 3 days at 25°C, a compact sediment is present, but a pellicle is absent. Following 1–3 weeks of incubation short pseudohyphal and septate hyphal fragments are present. The septate hyphae may bear clamps (or pseudoclamps) (Figure 2b) and the cells of the pseudohyphae may be connected by curved buds reminiscent of clamps of dikaryotic hyphae. After 3 weeks an incomplete climbing pellicle is present. On Dalmau plate culture on corn meal agar after 7 days at 25°C, pseudohyphae and true hyphae are absent, and conjugation tubes are present. Following prolonged incubation on several agar media, including 2% malt extract, corn meal, potato dextrose and yeast extract-malt extract-peptone-glucose agars, characteristic curved buds are interconnecting some cells. The connected cells may be independent (Figure 2c.1) or mother cell-bud pairs (Figure 2c.2). In addition, conjugating tubes and conjugations were also observed. The conjugation tubes may be simple (Figure 2d) or twisting and occasionally are ramified (Figure 2e). Formation of basidia and basidiospores was not observed even after 10 weeks incubation. The additional media acetate agar, glucose-peptone-yeast extract agar and vegetable juice agar (V8) were also applied to try to induce sexual state following incubation at 25°C for 10 weeks. The formation of conjugation tubes was abundant upon isolation of the strain, but has decreased during maintenance after subsequent subculturing. The formation of the conjugation tubes could be stimulated by incubating the cultures at lower temperatures (6°C or 15°C) and could be fully restored by freezing and thawing the strain in liquid nitrogen (i.e. freezing in 10% glycerol, direct immersion in nitrogen without stepwise cooling, thawing after 1 day in 37°C water bath). Ballistoconidia are not produced. Physiological/biochemical test responses can be seen in Table 2 and Table S1.

Strain investigated: CBS 12505^T^ ( = NRRL Y-48783^T^, NCAIM Y.01952^T^), from exudate of oak (*Quercus cerris*) in the Pilis Mountains near Visegrád, Hungary, isolated by G. Péter. The type strain has been deposited in the National Collection of Agricultural and Industrial Microorganisms in Budapest (Hungary), Centraalbureau voor Schimmelcultures (CBS) and ARS culture collections (NRRL).

## Supporting Information

Figure S1 **MSP-PCR fingerprinting of** ***Bandoniozyma complexa*** **representative strains: DNA banding patterns obtained with primer M13. M –1 kbp ladder, lane 1– group I CBS 11570^T^, lane 2– group I MA68d, lane 3– group II CBS 12531, lane 4– group III CBS 12398, lane 5– group III LD 2.09, lane 6– group III LD 3.02, lane 7– group III LR 3.11.**
(TIF)

Table S1 **Physiological/biochemical test responses of the newly proposed** ***Bandoniozyma*** **species.**
(DOC)

Information S1 **Details on the isolation methodology of** ***Bandoniozyma*** **strains.**
(DOC)

Information S2 **Physiological/biochemical test responses and sequencing analysis of the ITS region, EF1-alpha and mitochondrial cytochrome b genes of strains belonging to** ***Bandoniozyma complexa*** **.**
(DOC)
