# Supplementary figures and images for: Bandoniozyma gen. nov., a Genus of Fermentative and Non-Fermentative Tremellaceous Yeast Species
Source: PLoS One. 2012 Oct 9;7(10):e46060. doi: 10.1371/journal.pone.0046060 (PMC3467267; doi:10.1371/journal.pone.0046060)

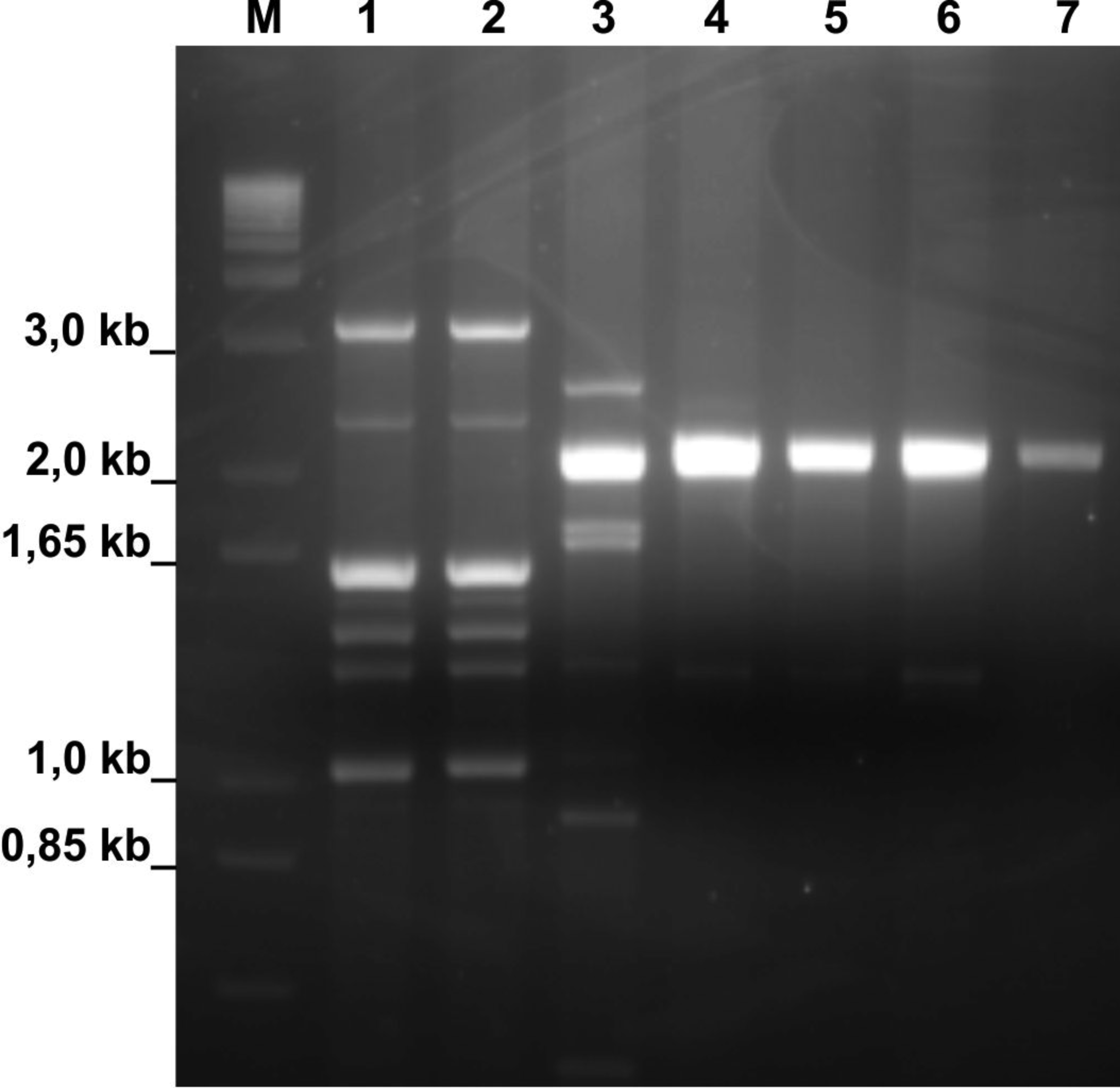

Supplement: Figure S1 — MSP-PCR fingerprinting of Bandoniozyma complexa representative strains: DNA banding patterns obtained with primer M13. M –1 kbp ladder, lane 1– group I CBS 11570T, lane 2– group I MA68d, lane 3– group II CBS 12531, lane 4– group III CBS 12398, lane 5– group III LD 2.09, lane 6– group III LD 3.02, lane 7– group III LR 3.11. (TIF) [file pone.0046060.s001.tif]
