# Supplementary material for: Bandoniozyma gen. nov., a Genus of Fermentative and Non-Fermentative Tremellaceous Yeast Species
Source: PLoS One. 2012 Oct 9;7(10):e46060. doi: 10.1371/journal.pone.0046060 (PMC3467267; doi:10.1371/journal.pone.0046060)
Supplement: Table S1 — Physiological/biochemical test responses of the newly proposed Bandoniozyma species. (DOC) [file pone.0046060.s002.doc]

Table S1: Physiological / biochemical test responses of the newly proposed *Bandoniozyma* species.

| Test responses* | *B. noutii* | *B. aquatica* | *B. complexa* | *B. fermentans* | *B. glucofermentans* | *B. tunnelae* ^a^ | *B. visegradensis* |
| --- | --- | --- | --- | --- | --- | --- | --- |
| Fermentation |  |  |  |  |  |  |  |
| D-Glucose | + | + | V | + | D | +,D | - |
| Galactose | - | - | - | - | - | - | - |
| Maltose | -,D | - | - | - | - | - | - |
| α-methyl-D-Glucoside | nd | nd | nd | - | - | nd | nd |
| Sucrose | + | nd | +^b^ | + | - | V | - |
| Trehalose | nd | nd | nd | + | - | nd | - |
| Melibiose | nd | nd | nd | - | - | nd | nd |
| Lactose | - | nd | nd | - | - | - | - |
| Cellobiose | nd | nd | nd | - | - | nd | nd |
| Melezitose | nd | nd | nd | - | - | nd | nd |
| Raffinose | + | nd | nd | - | - | V | - |
| Inulin | nd | nd | nd | - | - | nd | nd |
| Starch | nd | nd | nd | - | - | nd | nd |
| D-Xylose | nd | nd | -^b^ | - | - | nd | nd |
| Carbon sources |  |  |  |  |  |  |  |
| D-Glucose | + | + | + | + | + | + | + |
| D-Galactose | + | + | + | + | + | + | - |
| L-Sorbose | + | + | +,W | W | D | V | + |
| D-Glucosamine | - | - | - | - | D | - | - |
| D-Ribose | + | + | + | + | + | + | +,D |
| D-Xylose | + | + | + | + | + | + | + |
| L-Arabinose | + | + | + | + | + | + | - |
| D-Arabinose | + | - | +,D | W | + | + | +,D |
| L-Rhamnose | + | + | + | + | + | + | D |
| Sucrose | + | + | + | + | + | + | + |
| Maltose | + | + | + | + | + | + | + |
| α,α-Trehalose | + | +,W | +,W | + | + | + | + |
| α-methyl-D-Glucopyranoside | + | nd | + | W | + | + | + |
| Cellobiose | + | + | +,W | + | + | + | + |
| Salicin | + | + | V | - | + | + | + |
| Arbutin | + | nd | - | + | + | + | + |
| Melibiose | + | - | + | - | + | - | - |
| Lactose | - | + | V | - | -,W | V | - |
| Raffinose | + | - | + | - | + | V | - |
| Melezitose | + | + | + | + | + | + | + |
| Inulin | - | - | V | - | - | V | - |
| Starch | - | - | V | W | - | V | +,W |
| Glycerol | + | + | V | - | + | +,D | +,D |
| Erythritol | - | + | V | - | + | +,D | - |
| Ribitol | + | + | +,D | W | + | +,D | D,V |
| Xylitol | + | + | V | W | + | +,D | D,V |
| L-Arabinitol | + | + | V | + | + | +,D | - |
| D-Glucitol | + | + | + | + | + | + | + |
| D-Manitol | + | + | + | + | + | + | + |
| Galactitol | V | + | V | + | W | - | - |
| *myo*-Inositol | + | + | + | + | + | + | + |
| D-Glucono-1,5-lactone | V | nd | + | + | + | + | + |
| 2-Keto-D-gluconate | + | nd | + | + | + | + | + |
| 5-Keto-D-gluconate | nd | nd | + | + | nd | nd | nd |
| D-Gluconate | + | + | + | + | + | + | + |
| D-Glucuronate | + | nd | + | + | + | + | + |
| D-Galacturonate | - | nd | + | + | W | + | V |
| DL-Lactate | + | - | +,D | W | - | + | + |
| Succinate | + | + | +,D | W | + | + | + |
| Citrate | + | V | V | W | W | + | + |
| Methanol | - | - | - | - | - | - | - |
| Ethanol | + | + | +,W | W | W | + | + |
| Propane-1,2-diol | D | nd | -,W | W | + | - | - |
| Butane-2,3-diol | - | nd | - | - | - | - | - |
| Quinic acid | - | nd | nd | nd | - | - | nd |
| Saccharate | + | nd | +,W | nd | nd | + | - |
| Galactonate | + | nd | nd | nd | nd | D | nd |
| N-acetyl-D-glucosamine | nd | + | +,D | + | nd | nd | D |
| Hexadecane | nd | - | - | nd | nd | nd | - |
| Acetone | nd | - | - | nd | nd | nd | nd |
| Ethyl acetate | nd | - | - | nd | nd | nd | nd |
| Isopropanol | nd | - | - | nd | nd | nd | nd |
| Carbonate | nd | - | V | nd | nd | nd | nd |
| Nitrogen sources |  |  |  |  |  |  |  |
| Nitrate | - | - | - | - | - | - | - |
| Nitrite | +,W | - | V | W | - | - | W |
| Ethylamine | + | + | + | + | + | V | - |
| L-Lysine | + | + | +,D | + | + | + | + |
| Cadaverine | + | nd | + | + | + | + | W |
| Creatine | - | nd | - | - | - | - | - |
| Creatinine | - | nd | - | nd | - | - | - |
| D-Tryptophan | nd | nd | nd | nd | D | nd | nd |
| D- Glucosamine | - | nd | - | nd | - | - | - |
| Imidazole | - | nd | nd | nd | - | - | - |
| Vitamin requirements |  |  |  |  |  |  |  |
| Vitamin free | nd | + | + | + | - | - | - |
| w/o Thiamin | nd | nd | nd | nd | - | - | nd |
| w/o Biotin | nd | nd | nd | nd | + | + | nd |
| w/o Biotin and thiamin | nd | nd | nd | nd | - | - | nd |
| w/o pyridoxine and thiamin | nd | nd | nd | nd | - | - | nd |
| w/o pyridoxine | nd | nd | nd | nd | + | + | nd |
| w/o inositol | nd | nd | nd | nd | + | + | nd |
| w/o pantothenate | nd | nd | nd | nd | + | + | nd |
| w/o niacin | nd | nd | nd | nd | + | + | nd |
| w/o PABA | nd | nd | nd | nd | + | - | nd |
| w/o aminoacids | nd | + | + | nd | nd | nd | nd |
| Other tests |  |  |  |  |  |  |  |
| 25 ºC | + | + | + | + | + | + | + |
| 30 ºC | + | + | + | + | + | + | + |
| 35 ºC | V | - | + | + | nd | V | - |
| 37 ºC | - | - | V | - | - | - | nd |
| 40 ºC | nd | - | - | - | - | nd | nd |
| 42 ºC | nd | nd | - | - | - | nd | nd |
| 0.01% cycloheximide | + | + | V | + | + | V | + |
| 0.1% cycloheximide | + | nd | V | + | nd | - | + |
| 1% Acetic acid | - | - | - | - | - | nd | - |
| 50%D-Glucose | + | - | V | - | - | V | - |
| 60% D-Glucose | - | nd | - | - | - | - | nd |
| 10% NaCl/Glucose 5% | nd | + | + | + | W | V | - |
| 16% NaCl | nd | nd | - | - | - | - | nd |
| Urea hydrolysis | + | nd | + | + | + | + | + |
| Diazonium Blue B reaction | + | + | + | + | + | + | + |
| Starch formation | + | W | + | - | + | +,W | + |
| Acetic acid formation | - | nd | - | - | - |  | - |
| Tween20 | nd | nd | + | nd | nd | nd | nd |
| Tween80 | nd | nd | + | nd | nd | nd | nd |
|  |  |  |  |  |  |  |  |

*Test results: +, positive; D, delayed positive; W, weak; -, negative; V, variable; nd, not determined.

^a^ *Cryptococcus tunnelae* CBS 6123 splits fat (Eijkman test); does not grow on 1% acetic acid; does not produce acetic acid; CBS 6024 has a plasmid of 5kb (H. Fukuhara), CBS 6123 and CBS 8024 do not have plasmids [data from CBS website].

^b^ Results for *B. complexa* strain IMUFRJ 51948.
