# Supplementary material for: Bandoniozyma gen. nov., a Genus of Fermentative and Non-Fermentative Tremellaceous Yeast Species
Source: PLoS One. 2012 Oct 9;7(10):e46060. doi: 10.1371/journal.pone.0046060 (PMC3467267; doi:10.1371/journal.pone.0046060)
Supplement: Information S1 — Details on the isolation methodology of Bandoniozyma strains. (DOC) [file pone.0046060.s003.doc]

Text S1: Details concerning isolation of *Bandoniozyma* strains.

*Yeast isolation and maintenance*:

*Bandoniozyma noutii* (CBS 8364^T^ = DBVPG 4489^T^; CBS8365, CBS 8368) was collected from the exudate of the loquat tree, *Eriobotrya japonica* (Rosaceae), in the Tijuca forest in Rio de Janeiro, Brazil and from a flower of *Pimenta dioica* (Myrtaceaea) in Pau da Fome, Pedra Branca, RJ, Brazil.

The three strains of *Bandoniozyma tunnelae*, which were kept in CBS (CBS Fungal Biodiversity Center, The Netherlands) as CBS 8024^T^, CBS 6123 and CBS 6024, were isolated in Finland by E. Tunnela from unknown substrates [CBS 6024, 6123] and human nails [CBS 8024 ^T^]. Further details concerning the isolation of these strains are not available.

*Bandoniozyma visegradensis* strain CBS 12505^T^ was isolated from exudate of an oak tree (*Quercus cerris*) in Pilis Mountains, Hungary near the small town Visegrád, in May 2005 (spring season). The tree exudate was sampled with a sterile cotton swab. The swab was vortexed with 5 mL Yeast Nitrogen Base (YNB, Difco). Following serial dilution the sample was surface plated on Rose-Bengal Chloramphenicol (RBC) agar and incubated at 25°C in darkness, for 7 days. Individual colonies showing different appearance were picked and purified, and maintained on 2% malt extract agar slants at 6 °C.

*Bandoniozyma glucofermentans* CBS 10381^T^ and NRRL Y-48077 were isolated from the gut of beetles collected at Barro Colorado Island, Panama. The beetle gut was removed aseptically and transferred to 0.7% saline. Crushed gut solution was streaked on acidiﬁed YM agar (Difco YM broth, 2% plain agar, adjusted to pH 3.5 with HCl), and the plates were incubated at 25 ºC [1].

The strain of *Bandoniozyma complexa* CBS 12398 was isolated from pineapple purchased from a traditional market, Hsinchu, Taiwan, 2008, and the strain of *Bandoniozyma fermentans* CBS 12399^T^ was recovered from the fruiting body of an unidentified mushroom in a bamboo field in Beinan, Taitung, Taiwan, 2009. Isolation of strain CBS 12398^T^ was performed according to [2]. Approximately 1.0 g of the samples of fruit were cut into pieces and put into 9 mL of YM broth containing 0.005% of chloramphenicol and then vortex-mixed. One hundred microliter of successive decimal dilutions was spread on acidified YMA (1% glucose, 0.5% peptone, 0.3% yeast extract, 0.3% malt extract, 1.5% agar, pH 3.5). The plates were incubated at 24°C for 3 days. Representative colonies were picked and purified by streaking onto YM agar. The yeasts were grown on YM agar at 24°C for 3 days, followed by preservation in the freezer at -70 ^o^C. For isolation of strain CBS 12399^T^ from a mushroom, approximately 1.0 g of the samples of the fruiting body were cut into pieces and put into 9 mL of YNX broth (1.17% yeast nitrogen base, 0.5% xylose) containing 0.005% of chloramphenicol, then vortex-mixed and incubated at 25 ^o^C for 3 days. One hundred microliter of successive decimal dilutions was spread on YNX agar. The plates were incubated at 24 ^o^C for 3 days. Representative colonies were picked and purified by streaking onto YM agar. The yeasts were grown on YM agar at 24°C for 3 days, followed by preservation in the freezer at -70°C and/or on YMA at 4°C.

*Bandoniozyma aquatica* strain UFMG-DH-4.20^T^ (CBS 12527^T^) was isolated from freshwater in at Dom Helvécio lake from Parque Estadual do Rio Doce (Minas Gerais - Southeast Brazil), an Ecological Reserve of Atlantic Rain Forest, in July 2008, while *Bandoniozyma complexa* strains UFMG-LD 2.09 and UFMG-LD 3.02 were isolated from Lago de Dentro lake, and strain UFMG-LR3.11 from Lago Rico lake, both in Parque Estadual do Cantão (Tocantins - Northern Brazil), an Ecological Reserve of Amazon Forest, in July 2008. Water samples were filtered through sterile nitrocellulose membranes of 0.45 µm and 47-mm of diameter using a Nalgene® filtering device and vacuum pump. The membranes were placed on the surface of YMA (yeast extract 0.3%; malt extract 0.3%; peptone 0.5%; dextrose 1%; agar 2%; pH 4), containing 0.02% chloramphenicol. The strains were purified and maintained on agar slants at 4 ºC, covered with sterile mineral oil and/or stored by cryopreservation at -80°C.

*Bandoniozyma complexa* strain CBS 12531 was isolated from an aluminum screw with signs of corrosion from an energy transmission tower at Suzano/São Paulo state in Southeast Brazil. It was recovered in MSA medium (malt extract 3%, soy peptone 3%, agar 2%) at pH 4.0 and 28 °C, after a previous enrichment procedure using the same broth medium for 10 hours [3]. Strain IMUFRJ 51948 was isolated at the end of the rainy season in 2006, at Restinga de Maricá (22 57’ 37” S, 42 50’ 43” W), 30 Km to the east of Rio de Janeiro, Brazil. Healthy leaves of *Neoregelia cruenta* (Bromeliaceae) were sampled, and 11 g were cut into approximately 1 cm^2^ peaces, placed in an Erlenmeyer containing 100 mL of saline solution, and vigorously shaken for 10 minutes. 0.1 mL of this suspension was inoculated in BIL-VBC (glucose 0.5%, yeast extract 0.5%, ammonium sulfate 0.1%, monobasic sodium phosphate 0.2%, magnesium sulfate (7.H_2_O) 0.01%, potassium chloride 0.02%, bromocresol green 0.02%, agar 2%) plates containing 0.05% amoxicillin and 0.01% chloramphenicol.

*Bandoniozyma complexa* strains CBS 11570^T^ and MA68d were isolated in 2005 from air samples in a timber factory during the South Hemisphere winter (May) and summer (December) seasons, respectively, in Cachoeira do Sul/Rio Grande do Sul state in South Brazil (30º 00.470’ S 52º 54.865’ W). Plates containing YEPD agar (glucose 2 %, peptone 1 %, yeast extract 0.5 %, agar 2 %) supplemented with 0.03% chloramphenicol were maintained open in distinct points inside the timber factory. After an hour, the plates were closed and incubated at 30ºC for 10 days. The strains were purified, and maintained on agar slants at 4 ºC covered with sterile mineral oil.

1. Suh S-O, Blackwell M (2005) Four new yeasts in the *Candida mesenterica* clade associated with basidiocarp-feeding beetles. Mycologia 97: 170–180.

2. Liu YR, Huang LY, Young SS, Chang CF, Lee CF (2011) *Asterotremella meifongana* sp. nov. and *Asterotremella nantouana* sp. nov., two anamorphic basidiomycetous yeasts isolated from soil and mushrooms. Antonie van Leeuwenhoek 99: 643-650.

3. Sette LD, Passarini MRZ, Rodrigues A, Leal RR, Simioni KCM, Nobre FS, Brito BR, Rocha AJ, Pagnocca FC (2010) Fungal diversity associated with Brazilian energy transmission towers. Fung Div 44: 53–63.
