# Supplementary material for: Bandoniozyma gen. nov., a Genus of Fermentative and Non-Fermentative Tremellaceous Yeast Species
Source: PLoS One. 2012 Oct 9;7(10):e46060. doi: 10.1371/journal.pone.0046060 (PMC3467267; doi:10.1371/journal.pone.0046060)
Supplement: Information S2 — Physiological/biochemical test responses and sequencing analysis of the ITS region, EF1-alpha and mitochondrial cytochrome b genes of strains belonging to Bandoniozyma complexa . (DOC) [file pone.0046060.s004.doc]

Supporting Information S2: Physiological / biochemical test responses and sequencing analysis of the ITS region, EF1-alpha and mitochondrial cytochrome b genes of strains belonging to *Bandoniozyma complexa*.

Table: Physiological / biochemical test responses of the three *Bandoniozyma complexa* groups of strains.

| Test responses* | Group I | Group II | Group III |
| --- | --- | --- | --- |
| Fermentation |  |  |  |
| D-Glucose | + | + | - |
| Galactose | - | - | - |
| Maltose | - | - | - |
| α-methyl-D-Glucoside | nd | nd | nd |
| Sucrose | nd | +^a^ | nd |
| Trehalose | nd | nd | nd |
| Melibiose | nd | nd | nd |
| Lactose | nd | nd | nd |
| Cellobiose | nd | nd | nd |
| Melezitose | nd | nd | nd |
| Raffinose | nd | nd | nd |
| Inulin | nd | nd | nd |
| Starch | nd | nd | nd |
| D-Xylose | nd | -^a^ | nd |
| Carbon sources |  |  |  |
| D-Glucose | + | + | + |
| D-Galactose | + | + | + |
| L-Sorbose | nd | + | +,W |
| D-Glucosamine | nd | - | - |
| D-Ribose | + | + | + |
| D-Xylose | + | + | + |
| L-Arabinose | + | + | + |
| D-Arabinose | +,D | + | + |
| L-Rhamnose | + | + | + |
| Sucrose | + | + | + |
| Maltose | + | + | + |
| α,α-Trehalose | + | + | +,W |
| α-methyl-D-Glucopyranoside | + | + | + |
| Cellobiose | + | + | +,W |
| Salicin | + | +,D | V |
| Arbutin | nd | nd | - |
| Melibiose | + | + | + |
| Lactose | - | - | V |
| Raffinose | + | + | + |
| Melezitose | nd | + | + |
| Inulin | -,W | -,V | V |
| Starch | +,D | - | +,W |
| Glycerol | + | - | + |
| Erythritol | + | - | + |
| Ribitol | +,D | + | + |
| Xylitol | - | - | V |
| L-Arabinitol | + | - | V |
| D-Glucitol | + | + | + |
| D-Manitol | + | + | + |
| Galactitol | + | -,w | + |
| *myo*-Inositol | + | + | + |
| D-Glucono-1,5-lactone | nd | + | + |
| 2-Keto-D-gluconate | nd | + | + |
| 5-Keto-D-gluconate | nd | nd | + |
| D-Gluconate | + | + | + |
| D-Glucuronate | nd | + | + |
| D-Galacturonate | nd | nd | + |
| DL-Lactate | +,D | + | +,W |
| Succinate | +,D | + | +,W |
| Citrate | + | - | +,W |
| Methanol | - | - | - |
| Ethanol | nd | + | +,W |
| Propane-1,2-diol | nd | - | W |
| Butane-2,3-diol | nd | - | - |
| Quinic acid | nd | nd | nd |
| Saccharate | nd | +,W | nd |
| Galactonate | nd | nd | nd |
| N-acetyl-D-glucosamine | +,D | + | + |
| Hexadecane | nd | - | - |
| Acetone | nd | nd | - |
| Ethyl acetate | nd | nd | - |
| Isopropanol | nd | nd | - |
| Carbonate | nd | nd | V |
| Nitrogen sources |  |  |  |
| Nitrate | - | - | - |
| Nitrite | + | + | -,W |
| Ethylamine | + | + | + |
| L-Lysine | +,D | + | + |
| Cadaverine | nd | + | + |
| Creatine | - | nd | - |
| Creatinine | - | - | nd |
| D-Tryptophan | nd | nd | nd |
| D- Glucosamine | nd | - | nd |
| Imidazole | nd | nd | nd |
| Vitamin requirements |  |  |  |
| Vitamin free | nd | + | + |
| w/o Thiamin | nd | nd | nd |
| w/o Biotin | nd | nd | nd |
| w/o Biotin and thiamin | nd | nd | nd |
| w/o pyridoxine and thiamin | nd | nd | nd |
| w/o pyridoxine | nd | nd | nd |
| w/o inositol | nd | nd | nd |
| w/o pantothenate | nd | nd | nd |
| w/o niacin | nd | nd | nd |
| w/o PABA | nd | nd | nd |
| w/o aminoacids | nd | nd | + |
| Other tests |  |  |  |
| 25 ºC | + | + | + |
| 30 ºC | nd | nd | + |
| 35 ºC | nd | nd | + |
| 37 ºC | nd | nd | V |
| 40 ºC | nd | nd | - |
| 42 ºC | nd | nd | - |
| 0.01% cycloheximide | + | - | + |
| 0.1% cycloheximide | nd | - | + |
| 1% Acetic acid | nd | nd | - |
| 50%D-Glucose | + | + | V |
| 60% D-Glucose | nd | nd | - |
| 10% NaCl/Glucose 5% | + | + | + |
| 16% NaCl | - | nd | - |
| Urea hydrolysis | + | + | + |
| Diazonium Blue B reaction | + | + | + |
| Starch formation | + | + | + |
| Acetic acid formation | nd | nd | - |
| Tween20 | + | nd | nd |
| Tween80 | + | nd | nd |
|  |  |  |  |

*Test results: +, positive; D, delayed positive; W, weak; -, negative; V, variable; nd, not determined.

^a^ Results for *B. complexa* group II strain IMUFRJ 51948.

**Sequencing methodology**

**ITS parsimony network analysis**

ITS parsimony network analysis was carried out in TCS v. 1.21 (Clement et al. 2000) using 95% connection limit. Gaps were excluded from the alignments.

**Elongation factor 1 amplification and sequencing**

Nuclear DNA was extracted using a Biokit Genome DNA Extraction Kit (Biokit Co., Taiwan). The EF-1-α gene of the strain PL04 was amplified by PCR using the forward primers, EF1-728F(5' -CATCGAGAAGTTCGAGA GG-3') and EF1a-f597(5' -CGTCAGGACACACTGCAAATCTC-3'), and the reverse primers EF1-986R(5'-TACTTG AAGGAACCCTTACC-3), and EF1a-r1397(5'-GCAGTCAGCCTGGGAAGTAC-3') (Carbone and Kohn 1999; Nishi et al. 2010) with a Peltier thermal cycler (PTC-200, MJ Research). Sequencing reactions were performed with BigDye Cycler Sequencing Kit 4458688(Applied Biosystems) and analyzed on an ABI 3730XL DNA analyzer (Applied Biosystems).

**Mitochondrial cytochrome b amplification and sequencing**

Mitochondrial cytochrome *b* gene amplification was carried out using the forward primers E1M4 (5’-TGR GGW GCW ACW GTT ATTACT A-3’) and CytBf (5’-TAA CAA TCA CCA TCT AC-3’) and the reverse primer E2mr3 (5’-GGW ATA GCA CGT ARA AYW GCR TA-3’) according to Biswas et al. (2001) and Wang and Bai (2008). PCR amplicons were purified using *illustra PCR DNA and Gel Band Purification Kit* (GE Healthcare UK Limited, Buckinghamshire, UK). Sequencing reactions were performed in a 10 μl reaction using BigDye® Terminator Cycle Sequencing Kit v3.1 (Applied Biosystems) and analyzed on an ABI 3130 Genetic Analyzer (Applied Biosystems).

**Results and discussion**

Parsimony network analysis of the ITS sequences placed *Bandoniozyma complexa* groups I, II and III in the same network. Although Posada and Crandall (2001) state that sequences connected in a network represent alleles of a locus within the same species, this result only corroborates our preliminary conclusion that ITS sequences do not clearly differentiate the groups of strains within this species. Thus, a multigene sequencing approach was attempted using elongation factor 1 (EF1-alpha) and mitochondrial cytochrome b genes. Unfortunately, EF1-alpha gene sequencing was only successful for group III (strain CBS 12398), and mitochondrial cytochrome b sequencing for groups I (strain CBS 11570). In face of these results, the three groups are presently described as members of the same species.

>CBS12398_EF-1-alpha

GAAATCACTGGTTCATCTGAATGATCCTGATATTGACACGTTTACCCCGTTGACCCCAATATCGCCGACATCACCGCCCGTCGTCGTTCAACCTAGACCACCCGTATATGCTGCCAGGCGAACACTGGAACGATTGTCGTTGGCGTATGGCGGGGGACTGTGGCCAGAGCTCAGTATTTGGGCTCCTGAGAGGCACGCAACCACGGGCAAAAAGAGGAGACTCAACAAAAAGAGCAGTTTGACGGACGACGCATATCACCTGGGCAGCGGAGCTGGCACTGGGGTCAAATTGACACCGTGAGTTGGCTGTGTGTTGAAGTCGAGCTGACATTGCCAGAATCTCAGAGAGCGACCACTACTTTCAACTTCACCCCACTCTTCCCTTCTCCATCATGAAAGTTTCCAGCCTTGCACGGGTGTACTTCT

>CBS11570_cytb

ACAATTACTTTCAAAACGAGTTGTATTCAATAATTTAATAATTATTGCTGAAAAAATAAAAATTATTTATCAGCAAACAAATAATCATAAATTGAAAGTAAATAATTTTTCTTTATTTGTTTCAACGATCATATATTTATCTCTAATAATCTAAAAATTTCTATATTTTTTTTTATTAGTTAAGAAATGATCTAATTTGATTAGTAATAATCAAAAAATTTTTTAATATTCTTTGTCGTTTGGGGTGGATTCAGTGTAAGTAATGCTACTCTAAATCGATTCTTCAGTCTTCACTTCGTTCTTCCATTTGTTCTAGCTGCTCTTACACTAGTTCACATGATGACACTTCACACACATGGTTCAAGTAACCCTCTTGGAATTTCAAGTAATAGTGATAAGCTGCCAATGCATCCTTACTTCATGTTTAAGGATCTAGTAACTATGTTCGTATTCTTCGGAGGTATCGTAGGTATTGTATGTTACGCACCTAATATGCTAGGTCACAGTGATAACTATATCCCTGCTAACCCTATGTCAA

References:

Biswas SK, Yokoyama K, Nishimura K, Miyaji M (2001). Molecular phylogenetics of the genus *Rhodotorula* and related basidiomycetous yeasts inferred from the mitochondrial cytochrome b gene*.* Int J Syst Evol Microbiol 51: 1191–1199.

Carbone I, Kohn LM (1999). A method for designing sets for speciation studies in filamentous ascomycetes. Mycologia 91:553-556.

Clement M, Posada D, Crandall K (2000). TCS: a computer program to estimate gene genealogies. Mol Ecol 9: 1657–1660.

Nishi O, Iiyama K, Yasunaga-Aoki C, Shimizu S (2010) Incongruence between EF-1α Phylogeny and Morphology of *Metarhizium majus* and *Metarhizium guizhouense* in Japan. Entomotech 34: 19-23.

Posada D, Crandall KA (2001). Intraspecific gene genealogies: trees grafting into networks. Trends Ecol Evol 16: 37–45.

Wang QM, Bai FY (2008). Molecular phylogeny of basidiomycetous yeasts in the *Cryptococcus luteolus* lineage (Tremellales) based on nuclear rRNA and mitochondrial cytochrome b gene sequence analyses: proposal of *Derxomyces* gen. nov. and *Hannaella* gen. nov., and description of eight novel *Derxomyces* species. FEMS Yeast Res 8: 799–814.
